# β-Alanine supplemented diets enhance behavioral resilience to stress exposure in an animal model of PTSD

**DOI:** 10.1007/s00726-015-1952-y

**Published:** 2015-03-11

**Authors:** Jay R. Hoffman, Ishay Ostfeld, Jeffrey R. Stout, Roger C. Harris, Zeev Kaplan, Hagit Cohen

**Affiliations:** 1 Institute of Exercise Physiology and Wellness, Sport and Exercise Science, University of Central Florida, Orlando, FL 32816 USA; 2 Israel Defense Force, Medical Corps, Tel Hashomer, Israel; 3 Junipa Ltd, Newmarket, UK; 4 Anxiety and Stress Research Unit, Beer-Sheva Mental Health Center, Faculty of Health Sciences, Division of Psychiatry, Ben-Gurion University of the Negev, Beer-Sheva, Israel

**Keywords:** Supplementation, Carnosine, Military, Nutrition, BDNF, Corticosterone

## Abstract

This study investigated the effects of β-alanine (BA) ingestion on the behavioral and neuroendocrine response of post-traumatic stress disorder (PTSD) in a murine model. Animals were fed a normal diet with or without (PL) BA supplementation (100 mg kg^−1^) for 30 days. Animals were then exposed to a predator-scent stress (PSS) or a sham (UNEX). Behaviors were evaluated using an elevated plus maze (EPM) and acoustic startle response (ASR) 7 days following exposure to the PSS. Corticosterone concentrations (CS), expression of brain-derived neurotrophic factor (BDNF), and brain carnosine concentrations were analyzed a day later. Animals in PSS+PL spent significantly less time in the open arms and in the number of entries in the EPM than PSS+BA, UNEX+BA, or UNEX+PL. Animals in PSS+BA had comparable scores to UNEX+BA. Anxiety index was higher (*p* < 0.05) in PSS+PL compared to PSS+BA or animals that were unexposed. ASR and freezing were greater (*p* < 0.05) in animals exposed to PSS compared to animals unexposed. CS expression was higher (*p* < 0.05) in animals exposed to PSS compared to unexposed animals. Brain carnosine concentrations in the hippocampus and other brain sections were significantly greater in animals supplemented with BA compared to PL. BDNF expression in the CA1 and DG subregions of the hippocampus was lower (*p* < 0.05) in animals exposed and fed a normal diet compared to animals exposed and supplemented with BA, or animals unexposed. In conclusion, BA supplementation in rats increased brain carnosine concentrations and resulted in a reduction in PTSD-like behavior, which may be mediated in part by maintaining BDNF expression in the hippocampus.

## Introduction

Stress resulting from an acute traumatic experience can result in a variety of manifestations that include recurring and unwanted recollections or dreams of the event that cause significant behavioral changes (American Psychiatric Association 2013). Responses from acute stress may include avoidance of feelings or reminders of the event, marked arousal including irritability, hypervigilance, an elevated startle response, a concentration deficit or emotional numbing (American Psychiatric Association 2013). Although these responses to acute stress are often used to diagnose post-traumatic stress disorder (PTSD) (American Psychiatric Association 1994), several studies have indicated that some individuals are able to adapt within a short time period following the traumatic experience and not experience PTSD (Bisson et al. 2004; Bryant et al. 2008). Bryant et al. (2014) demonstrated that identification of an acute stress disorder within the first month of the traumatic experience appears to be only moderately sensitive (51 and 45 % within 3 and 12 months, respectively) for predicting PTSD. However, the absence of acute stress disorder appears to be highly predictive (95 and 94 % within 3 and 12 months, respectively) that the individual will not develop PTSD (Bryant et al. 2014).

The pathophysiology of PTSD is thought to be related to changes in the structure of neurons within areas of the brain that control stress and memory (McEwen 2007). The hippocampus is one of the most sensitive and adaptable regions of the brain, and many of the changes within the hippocampus occur within the dentate gyrus (DG)–CA3 region (McEwen 2007). The DG–CA3 region is thought to play a role in the memory of sequences of events, and several changes have been suggested to occur in this region during stress such as a suppression of neurogenesis or cell survival, and a degeneration of dendrites and synapses (McEwen 1999; Sousa et al. 1998; Stewart et al. 2005). The effect of stress on brain function though is not limited to the hippocampus only. The hippocampus works synergistically with the cortex and amygdala to control the processing of emotional memories (Richardson et al. 2004) and reduction of fear (Milad and Quirk 2002). Although the mechanism that is stimulating the structural remodeling of neurons in the brain is quite complex, evidence is compelling that changes in circulating glucocorticoids (McEwen 2007; Myers et al. 2014) and expression of brain-derived neurotrophic factor (BDNF) (Yao et al. 2011) are closely linked to the plasticity of brain function.

Stress has a profound influence on changes in the neuroendocrine system resulting in a significant elevation in glucocorticoids (Myers et al. 2014). Glucocorticoids are released from the adrenal gland and cross the blood–brain barrier to bind to receptors in neurons or glia cells. Glucocorticoid receptors are found in high concentrations within the brain, specifically in areas that are related to sites of stress such as the hippocampus, amygdala, and frontal cortex (Fuxe et al. 1987; Myers et al. 2014). Corticosterone injections in the dorsal hippocampus of rodents have been demonstrated to cause PTSD-like memory impairments that accompany impaired hippocampal function (Kaouane et al. 2012). Elevations in glucocorticoids are generally associated with dendritic remodeling and memory that often resemble what is seen during chronic stress (Miller and McEwen 2006). However, the glucocorticoid response to stress appears to follow an inverted U pattern. Very low or very high concentrations of circulating glucocorticoids during periods of stress are more likely to negatively alter neural plasticity (Miller and McEwen 2006).

BDNF is part of the neurotrophin family and has been demonstrated to have an important role in neuronal remodeling and modulating synaptic plasticity and neurotransmitter release (Castrén and Rantamäki 2010). Angelucci et al. (2014) recently compared BDNF concentrations in individuals diagnosed with PTSD to individuals who were exposed to a traumatic event but not diagnosed with PTSD. Serum BDNF concentrations were significantly lower in PTSD patients compared to the control subjects. In a rodent model, exposure to stress has been shown to down-regulate BDNF mRNA expression (Kozlovsky et al. 2007). Increasing BDNF expression, or decreasing glucocorticoid levels in individuals who are experiencing stress, may provide increased resiliency to PTSD following exposure to trauma.

Treatment for PTSD is quite varied and generally involves a combination of psychotherapy and pharmacological options that occur following diagnosis (Kirkpatrick and Heller 2014). However, less information is available regarding potential options for preventing or increasing resiliency to PTSD. Murakami and Furuse (2010) reported that a β-alanine supplemented diet in mice was able to increase brain carnosine concentrations in the cerebral cortex and hypothalamus and increase the concentration of BDNF in the hippocampus. These changes were also accompanied by significantly greater activity of the mice in the open arms of an elevated plus-maze test. Although β-alanine is considered to act as an inhibitory neurotransmitter that can cross the blood–brain barrier (Takeuchi et al. 2000), the lack of any significant change in β-alanine concentrations in the cerebral cortex and hypothalamus suggests that the anxiolytic effects observed are likely related to an elevation in brain carnosine (β-alanyl-l-histidine) concentrations. This is supported by others demonstrating that elevated brain carnosine can induce antidepressant-like activity (Tomonaga et al. 2004, 2008).

In consideration that β-alanine can increase brain carnosine concentrations, which may subsequently induce antidepressant activity, the primary purpose of this study was to examine the effect of 30 days of β-alanine ingestion on PTSD-like behavioral changes in rodents exposed to a predator-scent stress (PSS). A secondary purpose was to investigate the mechanisms underlying the potential beneficial effects of β-alanine ingestion by examining circulating corticosterone concentrations and BDNF expression in the hippocampus.

## Methods

### Animals

Adult male Sprague–Dawley rats weighing 200–250 g (*n* = 122) were habituated to housing conditions for at least 7 days. All animals were housed four per cage in a vivarium with stable temperature and a reversed 12-h light/dark cycle, with unlimited access to food and water. β-Alanine was provided with glucomannan in a powder form in an 80:20 blend. Rats were provided with 100 mg of the powder per kg of body mass (a total of 30 mg of powder was dissolved in 25 mL of water). PL-treated rats were provided with the vehicle (glucommanan) at the same relative dose. Animals were handled once daily. All testing was performed during the dark phase in dim red light conditions. This study was performed according to the principles and guidelines of the National Institute of Health Guide for the Care and Use of Laboratory Animals. All treatment and testing procedures were approved by the Animal Care Committee of the Ben-Gurion University of the Negev, Israel.

### Experimental design

Rats were randomly assigned to one of four treatment groups (*n* = 30 or 31 per group):

1. Unexposed and vehicle-treated group (UNEXP+PL): rats were fed regular food and water for 30 days and were exposed to fresh, unused litter for 15 min.
2. Unexposed and treated with β-alanine (UNEXP+BA): rats were provided β-alanine in their water and were exposed to fresh, unused litter for 15 min.
3. Exposed and vehicle-treated (EXP+PL): rats were fed regular food and water for 30 days and were exposed to PSS for 15 min.
4. Exposed and treated with β-alanine (EXP+BA): rats were provided β-alanine in their water exposed to PSS.

Following the 7-day acclimation period in which all rats received a normal powder diet, they were randomized into four groups. Following 30 days of either normal diet or β-alanine supplemented diet the rats were exposed to either the PSS or sham protocol. All behavioral tests were conducted 7 days following the PSS or sham protocol, and the rats were then sacrificed 24 h later and brains were removed. Diets were maintained until the end of the study. The validity of this model has been demonstrated in several studies (Cohen et al. 2004, 2012b; Kozlovsky et al. 2007).

### Predator-scent stress (PSS)

Following the 30-day supplementation regimen, animals were exposed to the PSS protocol. The PSS protocol consisted of placing the experimental animal on well-soiled cat litter (in use by the cat for 2 days, sifted for stools) for 10 min in a closed environment. Control animals were exposed to fresh, unused litter for the same amount of time. The situational reminder consisted of placing animals on fresh, unused cat litter for 15 min.

### Assessment schedule

Behavioral responses were assessed in the elevated plus-maze, acoustic startle response, and contextual freezing. All results were recorded and analyzed using an EthoVision automated tracking system (Noldus Information Technology, The Netherlands). Performance in the elevated plus-maze and acoustic startle response occurred 7-days following the initial exposure to the PSS. The contextual freezing measures were performed on day 8 following initial exposure. The delay in performing these measures from the PSS is based upon findings that extreme behavioral changes, which remain constant after 7 days of exposure and represent ‘chronic symptoms’ (Cohen et al. 2004) which persist over a prolonged duration (Cohen and Zohar 2004; Cohen et al. 2004). Following behavioral assessments, all animals were killed and their brains removed for analysis.

### Behavioral measures

#### Elevated plus-maze (EPM)

Behavioral assessments performed in the EPM have previously been described (Cohen et al. 2003, 2012a, b). The EPM is a plus-shaped platform with two opposing open and two opposing closed arms (open only towards the central platform and surrounded by 14-cm high opaque walls on three sides). Rats were placed on the central platform facing an open arm and allowed to explore the maze for 5 min. Each session was videotaped and subsequently scored by an independent observer. Arm entry was defined as entering an arm with all four paws. The following behaviors were assessed: time spent (duration) in open and closed arms and on the central platform; number of open and closed arm entries; and total exploration (entries into all arms). Total exploration was calculated as the number of entries into any arm of the maze to distinguish between impaired exploratory behavior, exploration limited to closed arms (avoidance), and free exploration. “Anxiety Index”, an index that integrates the EPM behavioral measures, was calculated as follows:

$$ {\text{Anxiety}}\,{\text{Index = 1}} - \left[ {\frac{{\left( {\frac{{{\text{time}}\,{\text{spent}}\,{\text{in}}\,{\text{the}}\,{\text{open}}\,{\text{arms}}}}{{{\text{total}}\,{\text{time}}\,{\text{on}}\,{\text{the}}\,{\text{maze}}}}} \right){ + }\left( {\frac{{{\text{number}}\,{\text{of}}\,{\text{entries}}\,{\text{to}}\,{\text{the}}\,{\text{open}}\,{\text{arms}}}}{{{\text{total}}\,{\text{exploration}}\,{\text{on}}\,{\text{the}}\,{\text{maze}}}}} \right)}}{ 2}} \right]. $$

Anxiety Index values range from 0 to 1 where an increase in the index expresses increased anxiety-like behavior.

#### Acoustic startle response

Startle response was measured using two ventilated startle chambers (SR-LAB system, San Diego Instruments, San Diego, CA). The SR-LAB calibration unit was used routinely to ensure consistent stabilimeter sensitivity between test chambers and over time. Each Plexiglas cylinder rests on a platform inside a sound-proofed, ventilated chamber. Movement inside the tube is detected by a piezoelectric accelerometer below the frame. Sound levels within each test chamber are measured routinely using a sound level meter to ensure consistent presentation. Each test session started with a 5-min acclimatization period to background white noise of 68 dB, followed by 30 acoustic startle trial stimuli in six blocks (110 dB white noise of 40 ms duration with 30 or 45 s inter-trial interval). Behavioral assessment consisted of mean startle amplitude (averaged over all 30 trials) and percent of startle habituation to repeated presentation of the acoustic pulse. Percent habituation—the percent change between the response to the first block of sound stimuli and the last—was calculated as follows:

$$ {\text{Percent}}\;{\kern 1pt} {\text{Habituation}}\;{\kern 1pt}  = \;{\kern 1pt} 100\; \times \;\left[ {\frac{{\left( {{\text{average}}\;{\kern 1pt} {\text{startle}}\;{\kern 1pt} {\text{amplitude}}{\kern 1pt} \;{\text{in}}\;{\kern 1pt} {\kern 1pt} {\text{Block}}\;{\kern 1pt} 1} \right)\; - \;\left( {{\text{average}}\;{\kern 1pt} {\text{startle}}\;{\kern 1pt} {\text{amplitude}}\;{\kern 1pt} {\text{in}}\;{\kern 1pt} {\kern 1pt} {\text{Block}}{\kern 1pt} \;6} \right)}}{{{\text{average}}{\kern 1pt} \;{\text{startle}}\;{\kern 1pt} {\text{amplitude}}{\kern 1pt} \;{\text{in}}\;{\kern 1pt} {\kern 1pt} {\text{Block}}{\kern 1pt} \;1}}} \right]. $$

### Contextual freezing measurement

Freezing behavior was scored during the situational reminder/cue exposure and was defined as an absence of all movement (except for respiration) (Kim et al. 1992). Total cumulative freezing time (total seconds spent freezing during each assessment period) was measured and calculated as a percentage of total time. Freezing behavior was recorded using an overhead video camera and scored for immobility using the recorded images. The videotape and the recorded images were both scored by a trained observer unaware of the treatment conditions.

### Blood sampling

Twenty rats from each group were decapitated with a guillotine. Care was taken to minimize situational stress: the area was thoroughly cleaned between each killing and bodies were removed. Trunk blood was collected and left at room temperature for 2 h and then centrifuged (1000*g* for 10 min at 4 °C) with a Hermle centrifuge. Serum (approximately 1 mL from each rat) was collected and stored at −80 °C until the analysis was performed.

### Measurement of serum corticosterone

Serum corticosterone was measured with ELISA Test Kit (Endocrine Technologies Inc. Newark, CA) according to the instructions of the manufacturer. Plates were read at 450 nm. All reactions were determined in duplicate. Inter-assay variation was 6.1 % and intra-assay variation 5.9 %. All samples were measured in duplicate. The lowest level detectable in this assay is 0.1 ng/mL of serum.

### Brain BDNF levels

#### Tissue preparation

Twenty-four hours following the behavioral tests, ten animals from each group were deeply anesthetized (ketamine and xylazine mixture) and perfused transcardially with cold 0.9 % physiological saline followed by 4 % paraformaldehyde (Sigma-Aldrich) in 0.1 M phosphate buffer (pH 7.4). Brains were quickly removed, postfixed in the same fixative for 12 h at 4 °C, and cryoprotected overnight in 30 % sucrose in 0.1 M phosphate buffer at 4 °C. Brains were frozen on dry ice and stored at −80 °C. Serial coronal sections (10 µm) at the level of dorsal hippocampus were collected for each animal, using a cryostat (Leica CM 1850) and mounted on coated slides.

#### Immunofluorescence

Sliced sections were air dried and incubated in frozen methanol (2 min) and in 4 % Para-formal-aldehyde (4 min). After three washes in phosphate-buffered saline (PBS) containing Tween 20 (PBS/T) (Sigma-Aldrich), the sections were incubated for 60 min in a blocking solution in normal goat or horse serum in PBS and then overnight at 4 °C with the primary antibodies against BDNF (1:250 each; Abcam). After three washes in PBS/T, sections were incubated in DyLight-488 in PBS containing 2 % normal serum for 2 h. Sections were washed and mounted with mounting medium (Vectrastain Vector laboratories, USA). Control staining was performed in the absence of the primary antibodies. Additionally, secondary fluorescent labels were swapped to check cross-reactivity and sections were incubated without any primary antibodies to check for any non-specific binding of the secondary antibodies.

#### Quantification

A computer-assisted image analysis system (Leica Application Suite V3.6, Leica, Germany) was used for quantitative analysis of the immunostaining and 50× objective lens was employed to assess the number of BDNF-IR positive cells in the hippocampus, divided into three (counted separately) areas: CA1 subfield, CA3 subfield, and dentate gyrus (DG). The regions of interest were outlined and computer-aided estimation was used to calculate the number of BDNF-IR cells in the pyramidal layer of CA1 and CA3, and in the granular layer of the DG. Seven representative sections of the hippocampus were chosen (between Bregma −2.30 and Bregma −3.60) from each animal, from each group. The sections were analyzed by two observers blinded to the treatment protocol. Standard technique was used to estimate the number of BDNF cell profiles per unit area for each investigated hippocampal structure.

### Measurement of brain carnosine concentrations

Carnosine concentrations in brain homogenates were determined by Liquid Chromatographic/tandem mass spectrometric (LC/MS/MS) analysis according to previously published methods (Aldini et al. 2004). Brains were partially thawed on ice and six brain regions were sampled: cerebral cortex, hypothalamus, hippocampus, amygdala, olfactory bulb, and thalamus. Each sample was weighed and transferred into individual vials for further homogenization.

Brain samples were homogenized in saline (0.9 % NaCl; 100 mg mL^−1^) supplemented with 1 mM EDTA, using Polytron Homogenizer. Homogenates were stored at −20 ± 5 °C prior to LC/MS/MS analysis. Homogenates were spiked with Internal Standard (D3-L-Dopa) and de-proteinized by addition of 100 μL of 700 mM PCA to 250 µL sample. After 1 min of Vortex, samples were centrifuged at 13,200 RPM [15.7 Relative Centrifugal Force (RCF)] for 12 min. The supernatant was diluted with 100 µL of Mobile phase and injected. L-Carnosine purchased from Sigma was used to prepare standards for the calibration curve (in ppb). Samples were diluted × 5 prior to analysis. The concentration read from the standard curve was multiplied by the dilution factor and converted to ppm units.

### Statistical analyses

The statistical analyses were performed using a one-way analysis of variance (ANOVA). In the event of a significant F ratio, LSD post hoc analysis was used for pairwise comparisons. All data are reported as mean ± SD. Pearson’s product-moment correlation was used to determine selected bivariate correlations. An alpha level of *p* < 0.05 was used to determine statistical significance. Data were analyzed using SPSS v22 software (SPSS Inc., Chicago, IL).

## Results

### Elevated plus-maze

**Fig. 1:**
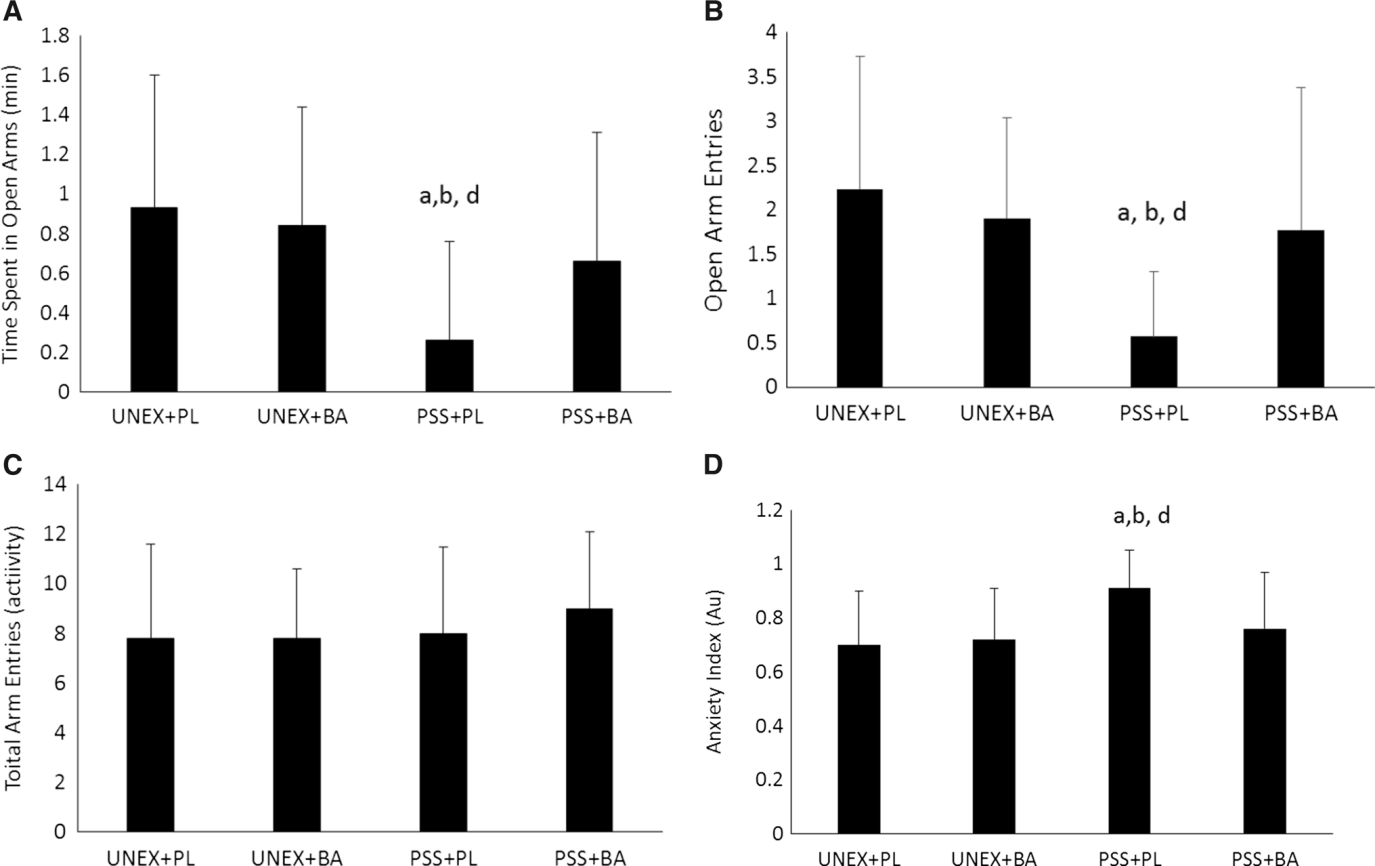
Behavioral performance in elevated plus-maze. **a** Comparison between the groups for time spent in open arms; **b** comparison between the groups in the number of open arm entries; **c** comparison between the groups in the total activity; **d** comparison between the groups in the anxiety-index; *a* significantly different than UNEX+PL, *b* significantly different than UNEX+BA, *d* significantly different than PSS+BA, *UNEX*+*PL* animals that were not exposed to the predator scent stress and fed a normal diet, *UNEX*+*BA* animals that were not exposed to the predator scent stress and supplemented with β-alanine, *PSS*+*PL* animals that were exposed to the predator scent stress and fed a normal diet, *PSS*+*BA* animals that were exposed to the predator scent stress and supplemented with β-alanine. All data reported as mean ± SD

Comparisons between the groups for time spent in open arms indicated a significant effect (*F* (3, 118) = 7.06, *p* < 0.001). Post-hoc analyses indicated that animals that consumed a normal diet and were exposed to PSS spent significantly less time in the open arms than all other treatment groups (*p* value ranging from <0.001 to 0.012). No other between-group differences were noted in time spent in open arms (see Fig. 1a). Comparisons of open arm entries (see Fig. 1b) also showed a significant interaction between groups (*F* (3, 118) = 9.55, *p* < 0.001). Normal diet fed animals that were exposed to PSS exhibited significantly (*p* value <0.001) fewer entries than all other treatment groups. (*p* value <0.001). No other group differences were noted in open arm entries.

Analysis of the total activity on the EPM of all groups (see Fig. 1c) revealed no significant differences between the groups (*F* (3, 118) = 0.925, *p* = 0.431). Based upon the integrated behavioral measures a significant difference in the anxiety-index was noted between the groups (see Fig. 1d) (*F* (3, 118) = 8.05, *p* < 0.001). Animals fed a normal diet and exposed to PSS exhibited significantly greater anxiety (*p* value ranging from <0.001–0.002) than other treatment groups (see Fig. 1d). No other between-group effects were noted.

### Acute startle response and startle habituation

**Fig. 2:**
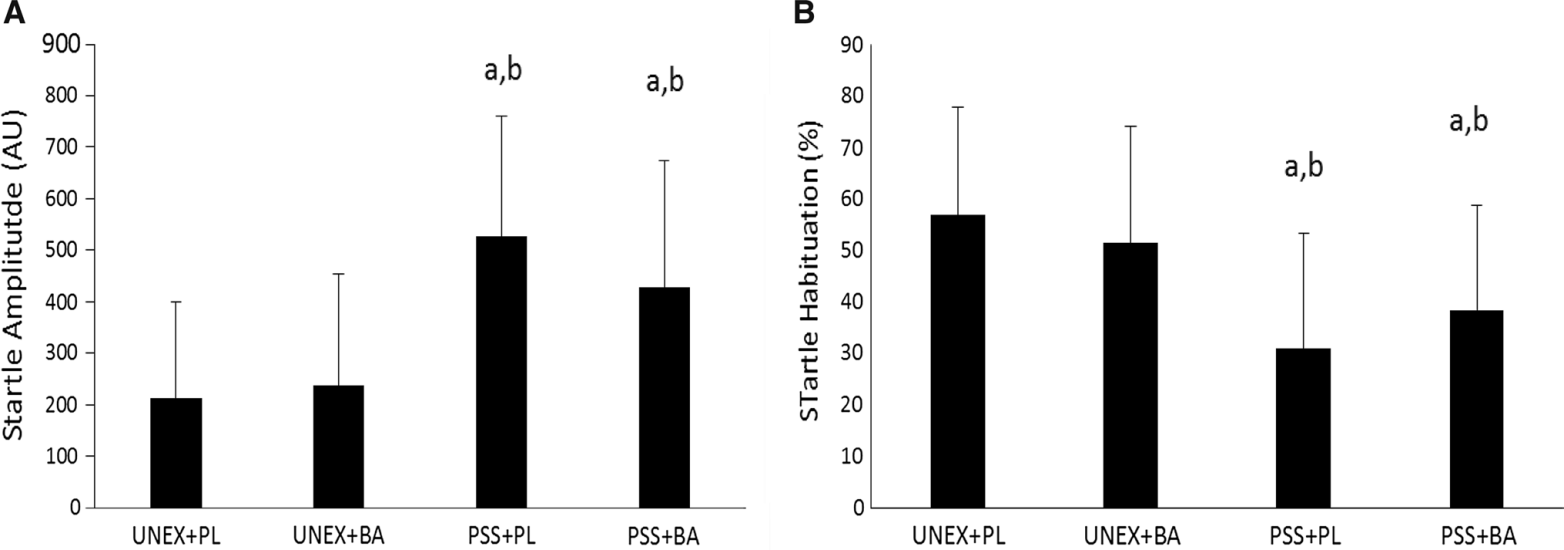
Acoustic Startle Response. **a** Comparisons between the groups in startle amplitude; **b** comparisons between the groups in startle habituation; *a* significantly different than UNEX+PL, *b* significantly different than UNEX+BA, *UNEX*+*PL* animals that were not exposed to the predator scent stress and fed a normal diet, *UNEX*+*BA* animals that were not exposed to the predator scent stress and supplemented with β-alanine, *PSS*+*PL* animals that were exposed to the predator scent stress and fed a normal diet, *PSS*+*BA* animals that were exposed to the predator scent stress and supplemented with β-alanine. All data reported as mean ± SD

Comparisons between the groups in acoustic startle amplitude revealed a significant group difference (*F* (3, 118) = 14.02, *p* < 0.001) (see Fig. 2a). Startle amplitude was significantly greater for animals that were exposed to PSS and fed a normal diet compared to unexposed animals, regardless of diet (*p* value <0.001), as well as for animals exposed to PSS and fed BA compared to unexposed animals fed a normal diet (*p* < 0.001) or supplemented with BA (*p* = 0.0001). In addition, animals that were exposed to PSS and whose diets were supplemented with BA tended to have a lower startle amplitude than exposed animals that were not fed BA (*p* = 0.085). A significant difference was also noted in startle habituation (*F* (3, 118) = 9.11, *p* < 0.001) (see Fig. 2b). Animals exposed to PSS regardless of diet had a significantly lower habituation score than all other groups (*p* value <0.001). No other significant differences between groups were noted.

### Effect of cue-exposure on freezing behavior at day 8

**Fig. 3:**
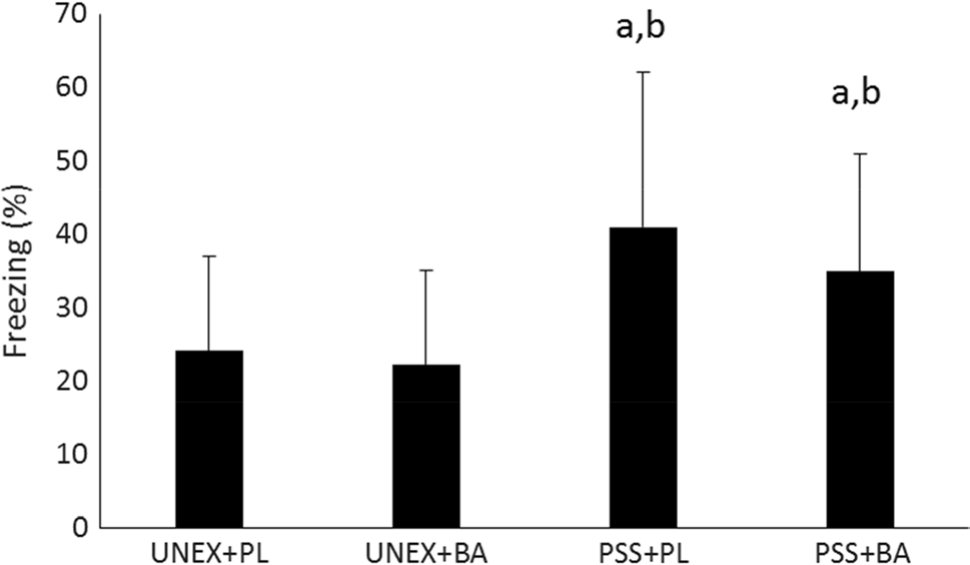
Freezing Behavior upon Cue-Exposure. *a* significantly different than UNEX+PL, *b* significantly different than UNEX+BA, *UNEX*+*PL* animals that were not exposed to the predator scent stress and fed a normal diet, *UNEX*+*BA* animals that were not exposed to the predator scent stress and supplemented with β-alanine, *PSS*+*PL* animals that were exposed to the predator scent stress and fed a normal diet, *PSS*+*BA* animals that were exposed to the predator scent stress and supplemented with β-alanine. All data reported as mean ± SD

Figure 3 depicts the freezing behavior upon cue-exposure. A significant difference between the groups was seen in freezing behavior (*F* (3118) = 9.342, *p* < 0.001). Animals exposed to PSS and fed a normal diet had significantly greater immobility upon cue than animals that were unexposed and fed a normal diet (*p* < 0.001) or fed a diet supplemented with BA (*p* < 0.001). Animals exposed to PSS and supplemented with BA were also observed to have significantly greater immobility upon cue than animals that were unexposed and fed a normal diet (*p* = 0.01) or fed a diet supplemented with BA (*p* = 0.003). Animals exposed to PSS and fed BA froze approximately 6 % less (40.9 % versus 34.8 %) less than animals exposed to PSS and fed a normal diet. However, this difference was not statistically different (*p* = 0.148).

### Corticosterone concentrations at day 8 post-PSS exposure

**Fig. 4:**
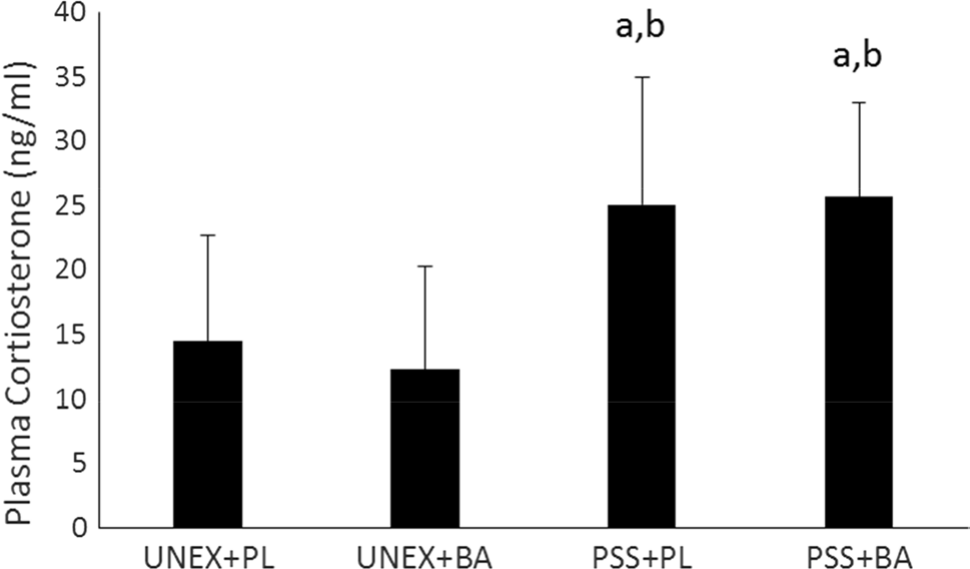
Corticosterone Concentrations on Day-8 Post-PSS Exposure. *a* significantly different than UNEX+PL, *b* significantly different than UNEX+BA, *UNEX*+*PL* animals that were not exposed to the predator scent stress and fed a normal diet, *UNEX*+*BA* animals that were not exposed to the predator scent stress and supplemented with β-alanine, *PSS*+*PL* animals that were exposed to the predator scent stress and fed a normal diet, *PSS*+*BA* animals that were exposed to the predator scent stress and supplemented with β-alanine. All data reported as mean ± SD

A significant difference between the groups (Fig. 4) was observed in corticosterone concentrations (*F* (3, 76) = 13.883, *p* < 0.001). Corticosterone concentrations in animals exposed to PSS and fed a normal diet were significantly greater than animals not exposed and fed a similar diet (*p* < 0.001) or supplemented with BA (*p* < 0.001). In addition, animals that were exposed to PSS and supplemented with BA were also observed to have significantly higher corticosterone concentrations than animals that were unexposed and fed a normal diet (*p* < 0.001) or were unexposed and supplemented with BA (*p* < 0.001). No significant differences (*p* = 0.837) were noted between exposed rats fed a normal diet compared to exposed rats supplemented with BA.

### BDNF expression on day-8 post-PSS exposure

**Fig. 5:**
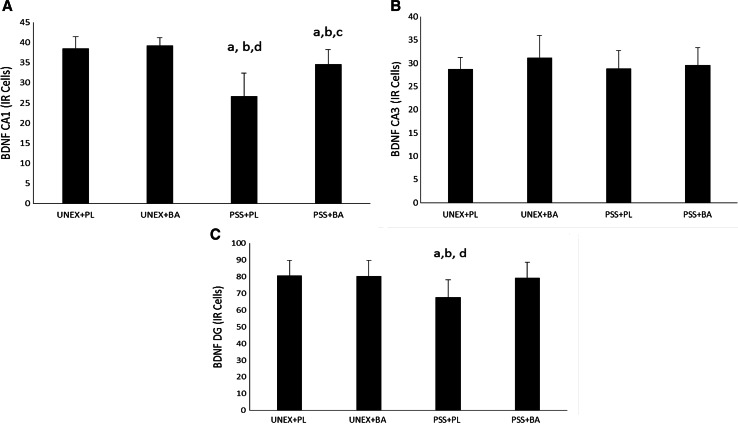
BDNF Expression on Day-8 Post-PSS Exposure. BDNF expression in the CA1 (**a**), CA3 (**b**) and DG (**c**) subregions. *a* significantly different than UNEX+PL, *b* significantly different than UNEX+BA, *c* significantly different than PSS+PL, *d* significantly different than PSS+BA, *UNEX*+*PL* animals that were not exposed to the predator scent stress and fed a normal diet, *UNEX*+*BA* animals that were not exposed to the predator scent stress and supplemented with β-alanine, *PSS*+*PL* animals that were exposed to the predator scent stress and fed a normal diet, *PSS*+*BA* animals that were exposed to the predator scent stress and supplemented with β-alanine. All data reported as mean ± SD

Comparisons between the groups in BDNF expression in the CA1, CA3, and DG subregions can be observed in Fig. 5a–c, respectively. Significant differences were noted in the CA1 (*F* (3, 36) = 23.2, *p* < 0.001) and DG subregions (*F* (1, 36) = 4.126, *p* = 0.013). No differences were observed between the groups in the CA3 subregion (*F* (1, 36) = 0.918, *p* = 0.442). BDNF expression in the CA1 and DG subregions in the animals that were exposed to PSS and fed a normal diet was significantly lower than that in all other groups (*p* values ranging from <0.001–0.011). In addition, BDNF expression in animals exposed to PSS and supplemented with BA was significantly lower than that in unexposed animals (*r* values ranging from 0.009–0.024), but significantly greater than that in exposed rats fed a normal diet in the CA1 (*p* < 0.001) only. In the DG subregion, BDNF expression in animals exposed to PSS but supplemented with BA were not statistically different from animals not exposed and either fed a normal diet (*p* = 0.765) or supplemented with BA (*p* = 0.818); however, the expression of BDNF in these animals exposed and fed BA was greater (*p* = 0.011) than that in animals exposed and fed a normal diet. No other differences were noted.

### Brain carnosine concentrations on day 8 post-PSS exposure

The concentration of carnosine in various areas of the brain can be observed in Table 1. Significant differences were noted in the hippocampus (*F* (3, 36) = 5.818, *p* = 0.002), cerebral cortex (*F* (1, 36) = 6.659, *p* = 0.001, hypothalamus (*F* (3, 36) = 5.348, *p* = 0.004), amygdala (*F* (3, 36) = 3.825, *p* = 0.018), and thalamus (*F* (3, 36) = 5.162, *p* = 0.005). No significant differences were noted in the olfactory bulb (*F* (3, 36) = 2.051, *p* = 0.124). In the hippocampus, cortex, hypothalamus, amygdala, and thalamus of animals that ingested BA and were exposed to PSS had significantly greater carnosine concentrations (*p* value ranging from <0.001–0.011) than animals who were fed a normal diet, regardless of exposure. Animals that were not exposed to PSS and supplemented with BA had a significantly greater carnosine concentration in the hippocampus (*p* = 0.030) and thalamus (*p* = 0.042) compared to animals not exposed and fed a normal diet. Additional comparisons between animals that were not exposed to PSS and supplemented with BA appeared to result in only strong trends (*p* value ranging from 0.051 to 0.089) when compared to animals fed a normal diet, regardless of exposure, in the cerebral cortex, hypothalamus, and amygdala. No differences were noted in comparisons between animals unexposed and fed BA compared to animals exposed and fed a normal diet in the hypothalamus (*p* = 0.113) and amygdala (*p* = 0.115) only.

**Table 1:** Brain carnosine concentrations (mg L^−1^)

	Hippocampus	Cortex	Hypothalamus	Amygdala	Olfactory bulb	Thalamus
UNEX+PL	0.71 ± 0.59	0.76 ± 0.79	0.64 ± 0.64	0.30 ± 0.34	11,011 ± 3910	0.77 ± 0.46
UNEX+BA	3.75 ± 3.98^a^	3.78 ± 4.47^a^	2.60 ± 2.85	2.34 ± 3.04	8309 ± 6887	3.99 ± 5.10^a^
PSS+PL	1.12 ± 0.90	0.96 ± 0.43	0.78 ± 0.46	0.55 ± 0.50	5744 ± 6491	1.16 ± 0.70
PSS+BA	5.58 ± 4.39^a, b^	6.55 ± 4.90^a, b^	4.54 ± 4.04^a, b^	3.52 ± 3.84^a, b^	13,109 ± 9773	5.97 ± 4.48^a, b^

All data reported as mean ± SD *UNEX*+*PL* animals that were not exposed to the predator scent stress and fed a normal diet, *UNEX*+*BA* animals that were not exposed to the predator scent stress and supplemented with β-alanine, *PSS*+*PL* animals that were exposed to the predator scent stress and fed a normal diet, *PSS*+*BA* animals that were exposed to the predator scent stress and supplemented with β-alanine

^a^significantly different than UNEX+PL

^b^significantly different than PSS+PL

## Discussion

The results of this study indicated that 30 days of β-alanine ingestion in rats was effective in attenuating some of the behaviors tested and associated with exposure to PSS. Rats fed a normal diet and exposed to PSS were observed to be significantly less active when placed in the elevated maze and had a greater anxiety level compared to animals that were either unexposed, or animals that were exposed and supplemented with BA. However, β-alanine supplementation to the diet was unable to alleviate all of the behaviors associated with exposure to stress. Animals exposed to PSS were shown to experience a significantly elevated startle response, freezing and a lower startle habituation than animals that were not exposed, regardless of whether they were supplemented with β-alanine or not. Nonetheless, animals that were exposed but supplemented with β-alanine demonstrated a 19 % lower startle response (*p* = 0.085) and a 15 % lower freezing response (*p* = 0.148) than animals exposed and which were not supplemented.

The animal model of PTSD used in this study has been previously established as a valid and effective method of examining biomolecular and physiological parameters of specific response patterns of stress (Cohen et al. 2012a; Matar et al. 2013). Although this model has not been previously used for β-alanine ingestion, it has been established as an effective approach to determine the effects of various interventions on the behavioral response to stress (Cohen et al. 2012a). In comparison to other studies, using this same stress model in rats, differences in the startle amplitude response (2.5 to 2.2 fold higher) seen between PSS+PL versus UNEX+PL or UNEX+BA, respectively, was consistent with the 2.5-fold increase previously reported by Cohen et al. (2004). Exposure to PSS in this study also resulted in a 30 % elevation in anxiety index and a nearly twofold increase in the freezing response compared to animals unexposed. These differences were similar to that reported by Cohen et al. (2012b), but lower in magnitude than that reported in the previous work by the same group (Cohen et al. 2004). The results of this study were also similar to other rodent models using a different PTSD model (Solanki et al. 2015). Those investigators using a single-prolonged stress model that incorporated psychological, physical, and pharmaceutical stresses also showed significant reductions in the activity patterns of rats in the EPM and a greater time of immobility or freezing when placed in a water tank.

Exposure to PSS was also accompanied by significant elevations in plasma corticosterone concentrations 7-days following the stress. This was consistent with other studies using the same stress model (Cohen et al. 2012b; Kozlovsky et al. 2007). Elevations in glucocorticoids are associated with exposure to stress (Myers et al. 2014), but recent evidence suggests that elevations in glucocorticoids may play a role in enhancing versus impairing recovery (Zohar et al. 2011). Previous research has indicated that a blunted hypothalamic-pituitary adrenal axis response to an acute stress may increase the susceptibility for PTSD-like symptoms (Cohen et al. 2006; McFarlane et al. 2011), which can be reversed with treatment of high-dose cortisone administration (Cohen et al. 2006; Kim et al. 2014; Zohar et al. 2011). However, glucocorticoid concentrations appear to demonstrate an inverted U effect. Although low concentrations may be a predictor for PTSD, high concentrations have also been suggested to be associated with morphological changes of neurons that also contribute to a negative behavioral response seen during stress (Sebastian et al. 2013). The hippocampus is sensitive to both acute and chronic stress. A chronic stress response may elevate glucocorticoid expression in the brain causing the neurons in the hippocampus to undergo reversible remodeling, especially in the DG-CA3 regions (McEwen 2007). These changes often involve dendritic atrophy (Romeo et al. 2004), which may result in diminished function in hippocampal-dependent memory tasks (Coburn-Litvak et al. 2003). In contrast, BDNF is reported to have the opposite effect on neuroplasticity by enhancing neurogenesis and dendritic remodeling (Yao et al. 2011). Elevations in corticosterone concentrations observed in both groups of rats exposed to PSS in this study were likely indicative of the stress experienced by those animals. However, the animals that were supplemented with BA appeared to recover or demonstrated a greater resiliency to stress in regard to their levels of anxiety and performance in the EPM. This may have been a function of both elevations in brain carnosine levels and an increased expression of BDNF. This is supported by the association seen in the increased expression of BDNF in the CA1 and DG subregions observed in this study with a reduction in the startle response (*r* = −0.341, *p* = 0.031 and *r* = −0.523, *p* = 0.001, respectively) and freezing response (*r* = −0.572, *p* < 0.001 and *r* = −0.335, *p* = 0.035, respectively) of the animals examined.

Rats supplemented with β-alanine in this study experienced significant elevations in carnosine concentrations in nearly all segments of the brain. Supplementation with β-alanine did not appear to have any influence on carnosine concentrations in the olfactory bulb. This was likely related to the high concentrations of carnosine generally seen in the olfactory bulb of most mammalian species (Bonfanti et al. 1999). However, differences in carnosine concentrations within the other brain regions support previous studies that showed β-alanine supplemented diets can increase brain carnosine concentrations in mice (Murakami and Furuse 2010) and chickens (Tomonaga et al. 2005, 2012). Tomonaga et al. (2008) suggested that brain carnosine can attenuate depression-like behavior by reducing brain metabolites of norepinephrine, or through stimulation of histaminergic neurons through one of its constituents; histidine. Others have suggested that carnosine might influence brain antioxidant activity (Kohen et al. 1988), while others have reported that elevations in brain carnosine concentrations in the hypothalamus and cerebral cortex are associated with increases in the concentration of BDNF in the hippocampus of mice (Murakami and Furuse 2010). In the present study, elevations in brain carnosine concentrations in the PSS+BA group were associated with maintaining expression of BDNF in the CA1 and DG subregions of the hippocampus compared to PSS+PL rats. Elevations in carnosine concentrations in the hippocampus, cortex, hypothalamus, amygdala++ and thalamus were inversely associated with anxiety index (*r* value ranging from −0.471 to −0.550, *p* value <0.002) and positively associated with improved time spent in the open arms (*r* value ranging from 0.453 to 0.521, *p* value <0.003).

The significant decrease in BDNF expression observed in rodents exposed to PSS and fed a normal diet is consistent with previous studies reporting an attenuation of BDNF expression and PTSD-like behavioral stress response (Kozlovsky et al. 2007; Zohar et al. 2011). Although β-alanine ingestion and subsequent carnosine synthesis in the brain did not appear to increase BDNF expression in unexposed rats, it did appear to maintain BDNF expression in those rats that were exposed to PSS. Similar expression of BDNF expression in both UNEX+BA and UNEX+PL suggested that elevations in brain carnosine levels were not directly responsible for altering BDNF expression. These results on the surface appear to contrast with those of Murakami and Furuse (2010) who showed significant elevations in BDNF concentrations in hippocampus homogenates following β-alanine ingestion in physically stressed mice. However, those investigators did not use a non-stressed group as a control. In the present study, when comparing both groups of animals that were exposed to PSS, and differing only in regard to whether supplemented with β-alanine, the results confirm the findings of Murakami and Furuse (2010). The mechanism of elevated brain carnosine and maintenance of BDNF expression in the hippocampus is not well understood, but it may be related to its role as a neural protectant through its action as an antioxidant (Kohen et al. 1988). Oxidative stress and inflammation in the brain have been demonstrated to cause the development and further exacerbation of PTSD (Wilson et al. 2013). Thus, carnosine’s role as an antioxidant may prevent the neurodegeneration associated with elevated glucocorticoids and indirectly presere BDNF expression. Further research is warranted to examine the role of carnosine and oxidative stress in brain tissue.

In summary, the results of this study indicate that 30-day of β-alanine supplementation appears to promote resiliency and/or recovery from PSS. The protective effects associated with elevations in brain carnosine appear to be related to a protection of BDNF expression in the hippocampus. The precise mechanism of how elevated carnosine concentrations support BDNF expression requires additional research. This appears to be the first study known to demonstrate a potential role of β-alanine as a dietary supplement for the treatment or prevention of PTSD.
